# Relationship between Dietary Habits and Control of Lipid Profiles in Patients with Dyslipidemia Using Pravastatin

**DOI:** 10.3390/nu13113784

**Published:** 2021-10-25

**Authors:** Seo Young Kang, Tae Hee Jeon, Keun-Sang Yum, Sung Sunwoo, Hyun-Young Shin, Dae Hyun Kim, Kiduk Kim, Jong Lull Yoon, Jae-Kyung Choi, Young Sik Kim

**Affiliations:** 1 International Healthcare Center, Asan Medical Center, Seoul 05505, Korea; sykang@amc.seoul.kr; 2 Department of Family Medicine, Central Veterans Hospital, Seoul 05368, Korea; thju1105@hanmail.net; 3 Department of Famliy Medicine, Uijeongbu St. Mary’s Hospital, The Catholic University of Korea, Uijeongbu 11765, Korea; yks6303@catholic.ac.kr; 4 Department of Family Medicine, Asan Medical Center, University of Ulsan College of Medicine, Seoul 05505, Korea; sws@amc.seoul.kr; 5 Department of Family Medicine, Myongji Hospital, Hanyang University College of Medicine, Goyang 10475, Korea; shy801117@gmail.com; 6 Department of Family Medicine, Keimyung University Dongsan Hospital, Keimyung University School of Medicine, Daegu 42601, Korea; dhkim@dsmc.or.kr; 7 Department of Family Medicine, Sun Medical Center, Daejeon 34811, Korea; yetclose@naver.com; 8 Department of Family Medicine, Hallym University Dongtan Sacred Heart Hospital, Hallym University College of Medicine, Hwaseong 18450, Korea; lull@hallym.ac.kr; 9 Department of Family Medicine, Konkuk University Medical Center, Konkuk University School of Medicine, Seoul 05030, Korea; cjk@kuh.ac.kr

**Keywords:** dietary habits, cholesterol, lipid, dyslipidemia

## Abstract

We investigated the association between dietary habits, evaluated using the modified Mini Dietary Assessment Index for Koreans (MDA), and lipid control among patients aged ≥20 years who had used pravastatin for dyslipidemia for 6 months. Participants were administered questionnaires regarding sociodemographic characteristics and lifestyle factors. Odds ratios and 95% confidence intervals for the control of low-density lipoprotein cholesterol (LDL-C), triglyceride (TG), and total cholesterol (TC) at 6 months for each category of the modified MDA items were calculated through multivariate logistic regression analysis. The odds for controlled LDL-C was higher among those who consumed cholesterol-rich foods <1 time/week (3.27, 1.25–8.57) than for those who did so ≥4 times/week. The odds for controlled TG was higher among those who always consumed dairy products (2.96, 1.36–6.44), ate protein-rich foods three times/day (2.94, 1.06–8.10), and had a regular eating schedule (3.02, 1.30–7.00) than among those who did not have any of these. The odds for controlled TC was higher among those with a regular eating schedule (3.47, 1.55–7.76) than among their counterparts. Patients with dyslipidemia should consume less cholesterols, consume more dairy and protein-rich foods, and follow a regular eating schedule to control lipid profiles.

## 1. Introduction

Dyslipidemia is an important risk factor for cardiovascular diseases [1]. Previous studies have consistently reported a graded relationship between high levels of low-density lipoprotein cholesterol (LDL-C) and increased risk of coronary artery disease, while people with heathy lifestyles and fewer risk factors for coronary artery disease, particularly those with well-controlled lipid levels, have a reduced risk of coronary artery disease [1,2,3]. In Korea, approximately two out of every five adults aged ≥20 years have dyslipidemia [4]. The prevalence has markedly increased over the past 10 years, along with changes in dietary habits and lifestyles. Specifically, the prevalence of dyslipidemia in adult men and women is reported to be 45.6% and 31.3% and that of hyper-LDL-cholesterolemia is reported to be 17.4% and 21.0%, respectively [4]. Therefore, it is important for clinicians to provide individualized guidelines to control cholesterol levels.

Healthy lifestyles are associated with the control of dyslipidemia. Lifestyle modifications, including weight reduction, smoking cessation, and increased physical activity, have demonstrated clear benefits in improving lipid profiles [5,6]. Nevertheless, the role of nutrition and changes in dietary habits are particularly important in the management of dyslipidemia. In general, reducing dietary trans-fat and saturated fat reduces total cholesterol (TC) and LDL-C levels, and reducing the total amount of dietary carbohydrates and taking n-3 polyunsaturated fat supplements reduce triglyceride (TG) levels [5,7,8,9]. For the prevention and treatment of dyslipidemia, the Korean dyslipidemia guidelines recommend limiting total fat, saturated fat, and n-6 fatty acid intake to ≤30%, <7%, and <10% of the total energy intake, respectively; avoiding trans-fatty acid intake; and limiting total carbohydrate and sugar intake to ≤65% and 10–20% of total energy intake, respectively [6]. Despite these clear guidelines, it is difficult to apply these nutrient intakes directly to patient counseling because each food contains different amounts of nutrients, and it is difficult to calculate the amount of nutrients on every occasion.

The Korea National Health Screening Program provides nutritional evaluation, which is based on the Mini Dietary Assessment Index for Koreans (MDA) [10,11]. The modified MDA consists of 11 questions that the general public can answer easily. It has advantages of assessing the intake of food elements that need to be encouraged or discouraged, and allows evaluation of eating habits. Although dietary guidelines for patients with dyslipidemia are well established, studies that use practical tools to investigate the association between dietary habits and control of cholesterol levels are limited. Therefore, in this study, we investigated the association between dietary habits, as evaluated using the modified MDA, and control of lipid profiles at 6 months among patients who were started on pravastatin treatment for dyslipidemia.

## 2. Materials and Methods

### 2.1. Study Population

Study participants were consecutively enrolled by nine family physicians from eight hospitals, between October 2018 and July 2020. Individuals aged ≥20 years and who had started using pravastatin for dyslipidemia were eligible. We defined dyslipidemia and prescribed pravastatin based on the 2018 Guidelines for the Management of Dyslipidemia in Korea [6]: LDL-C ≥ 160 mg/dL for low-risk group (patients with one major risk factor, excluding diabetes), LDL-C ≥ 130 mg/dL for the moderate-risk group (patients with two or more major risk factors, excluding diabetes), LDL-C ≥ 100 mg/dL for the high-risk group (patients with carotid artery disease, abdominal aneurysm, or diabetes), LDL-C ≥ 70 mg/dL for the very high-risk group (patients with coronary artery disease, ischemic stroke, transient ischemic attack, or peripheral artery disease). Major risk factors referred to age (male ≥45 years, female ≥55 years), family history of premature coronary artery disease, hypertension, smoking, and low high-density lipoprotein cholesterol (HDL-C) level (<40 mg/dL). High HDL-C (≥60 mg/dL) is considered a protective factor; thus, one risk factor was deducted in such cases. Furthermore, patients who had previously been diagnosed with dyslipidemia and had been taking lipid-lowering agents were considered to have dyslipidemia.

Patients who corresponded to these definitions of dyslipidemia were invited to participate in the study, and those who agreed were consecutively enrolled and were prescribed pravastatin. During the study periods, 353 participants were enrolled. Among them, we excluded 63, 65, and 59 participants with missing values for LDL-C, TG, and TC, respectively, at 6 months, leaving 284 participants for the final analysis.

All study participants read and signed the informed consent before participating in the study. The study was conducted in accordance with the Declaration of Helsinki, and the study protocol and the written informed consent form were approved by the institutional review board of Asan Medical Center (2018-0111).

### 2.2. Data Collection and Measurements

After enrollment, participants were administered standardized questionnaires regarding sociodemographic characteristics including age, sex, household income, and marital status and lifestyle factors including alcohol consumption, smoking status, physical activity, and dietary habits. Monthly household income was categorized as <2 million Korean Republic Won (KRW), 2–3.99 million KRW, 4–5.99 million KRW, and ≥6 million KRW, and marital status was categorized as married, divorced/widowed/separated, and single. Participants’ current alcohol consumption and smoking status were categorized as either “yes” or “no.” Physical activity was classified as low, moderate, and high, based on the definitions of the International Physical Activity Questionnaire Short-Form, a scale based on activity intensity and exercise time during the previous 7 days [12]. A high activity level was defined as achieving 1500 metabolic equivalent (MET)-min/week with ≥3 days of vigorous-intensity physical activity or 3000 MET-min/week with 7 days of any combination of walking, moderate-intensity activity, or vigorous-intensity activity. Moderate physical activity was defined as performance of vigorous activity for ≥20 min/day for ≥3 days or moderate-intensity activity, or walking for ≥30 min/day for ≥5 days or achieving 600 MET-min/week with ≥5 days of any combination of walking, moderate-intensity activity, or vigorous-intensity activity. Low physical activity was defined as not meeting the criteria for either high or moderate physical activity. Dietary habits were evaluated by the modified version of the MDA [10]. The MDA was initially developed in 2003 to evaluate the quality of the diet of Koreans, based on dietary guidelines and the food tower for Koreans [11]. The modified MDA consists of 11 questions, including those on four food elements which are encouraged (such as milk, protein, vegetables, fruits); four food elements which are discouraged (such as fried food, cholesterol, sugar, salt); and three questions on dietary schedule, variety, and eating out (Table S1). Response options for questions evaluating consumption of milk, protein, vegetables, fruits, sugar, and salt, and use of dietary schedule were “always”, “usually”, and “no”, and response options for questions evaluating frequency of fried food and cholesterol were “≥4 times/week”, “2–3 times/week”, and “<1 time/week”. For the question evaluating variety of food types, the response options were “5 types”, “4 types”, and “≤3 types”, and the response options for the question evaluating the frequency of eating out were “≥5 times/week”, “2–4 times/week”, and “<1 time/week”. Furthermore, participants were asked whether their parents or siblings have been diagnosed with coronary artery disease. For those whose parents or siblings had coronary artery disease, the age at the diagnosis of coronary artery disease in the parents/siblings was recorded. In addition, information about medical history and concomitant medications was collected.

Data on anthropometric variables and laboratory findings at the time of enrollment and at the 6-month follow-up were collected. Height and weight were measured, and body mass index (BMI) was calculated as body weight divided by the square of the height (kg/m^2^). BMI was categorized as <23 kg/m^2^, 23–24.9 kg/m^2^, and ≥25 kg/m^2^ [13]. Systolic blood pressure (SBP) and diastolic blood pressure (DBP) were measured with a sphygmomanometer. Blood samples were collected after fasting for at least 12 h, and values for fasting glucose, hemoglobin A1c (HbA1c), TC, TG, HDL-C, and LDL-C were collected. Six months after enrolment, compliance with taking pravastatin in the previous 6 months was evaluated. Compliance with taking statin was categorized as either <80% or ≥80%.

### 2.3. Definitions of the Control of Cholesterol Levels

Target LDL-C goals were LDL-C < 160 mg/dL for the low-risk group, LDL-C < 130 mg/dL for the moderate-risk group, LDL-C < 100 mg/dL for the high-risk group, and LDL-C < 70 mg/dL for the very high-risk group [6]. When participants’ LDL-C levels were within these ranges at the 6 month follow-up, they were considered to have met the LDL-C target. The optimal control of TG and TC at the 6-month follow-up were defined as TG < 150 mg/dL and TC < 200 mg/dL [6].

### 2.4. Statistical Analysis

We presented distributions of baseline characteristics of the total study participants and participants with controlled LDL-C, TG, and TC at 6 months. We used the chi-square test to compare the sociodemographic and lifestyle characteristics between participants with and without controlled LDL-C, TG, and TC at 6 months. We performed Student’s *t*-test to compare anthropometric variables and laboratory findings between participants with and without controlled LDL-C, TG, and TC at 6 months. We presented numbers and percentages of participants corresponding to each category of the modified MDA items to demonstrate distributions of dietary habits according to the control status of LDL-C, TC, and TC. To evaluate factors associated with the control of lipid profiles at 6 months, multivariate logistic regression analysis was performed. Odds ratios (ORs) and 95% confidence intervals (CIs) for the control of LDL-C, TG, and HDL-C at 6 months were calculated for each sociodemographic and lifestyle characteristic. To evaluate the association between dietary habits and control of cholesterol levels, ORs and 95% CIs for the control of LDL-C, TG, and TC at 6 months for each category of the modified MDA items were calculated through multivariate logistic regression analysis. All analyses were conducted with IBM SPSS Statistics for Windows version 23.0 (IBM Corp., Armonk, NY, USA). *p* < 0.05 indicated statistical significance.

## 3. Results

### 3.1. Baseline Characteristics of the Study Participants

Table 1, Table S2 and Table S3 show the baseline characteristics of the study participants. Among the 284 study participants, LDL-C, TG, and TC levels were controlled in 75.4% (214 participants), 68.3% (194 participants), and 65.6% (186 participants) at 6 months. The proportions of participants who met the target LDL-C goal were higher among women than among men. The proportions of participants who met the target LDL-C goal increased as BMI decreased. The proportions of participants who met the target LDL-C goal were higher among those without diabetes and among those who had ≥80% compliance for taking the statin than among their counterparts. Participants who met the target LDL-C goal had lower fasting glucose levels and higher HDL-C levels than those who did not (Table 1). The proportions of participants with TG < 150 mg/dL at 6 months were higher among women and married participants than among their counterparts. The proportions of participants with TG < 150 mg/dL at 6 months increased as BMI decreased. Participants with TG < 150 mg/dL at 6 months had lower TG levels and higher HDL-C levels than those who did not (Table S2). The proportions of participants with TC < 200 mg/dL at 6 months increased as age increased. The proportion of participants with TC < 200 mg/dL at 6 months was higher among those with hypertension and those who had ≥ 80% compliance for taking the statin than among their counterparts. Participants with TC < 200 mg/dL at 6 months had lower TC, HDL-C, and LDL-C levels than those who did not (Table S3).

### 3.2. Distribution of Dietary Habits According to Low-Density Lipoprotein Cholesterol (LDL-C), Triglyceride (TG), and Total Cholesterol (TC) Levels at 6 Months

Table 2 shows the distributions of each category of the modified MDA items according to LDL-C, TG, and TC levels at 6 months. The proportions of participants who met the target LDL-C goal increased as the frequency of consumption of cholesterol-rich foods decreased. The proportions of participants with TG < 150 mg/dL increased as the consumption of dairy products and fruits increased and as participants followed a more regular eating schedule. The proportions of participants with TC < 200 mg/dL also increased when participants had a regular eating schedule.

### 3.3. Factors Associated with the Control of Lipid Profiles at 6 Months

Table 3 shows the ORs and 95% CIs for the control of LDL-C, TG, and TC at 6 months according to each variable. After adjusting for age, sex, household income, and marital status, the odds for achieving target LDL-C were higher among women (OR 2.46, 95% CI 1.29–4.68), those with BMI < 23 kg/m^2^ (2.47, 1.00–6.12), those with diabetes (6.70, 3.24–13.87), and those who had ≥80% of compliance in taking pravastatin (7.27, 1.85–28.58) than among their counterparts. The odds for having controlled TG at 6 months were higher among women (3.08, 1.63–5.82) than among men and among married participants (8.01, 1.40–45.92) than among single participants. The odds for having controlled TC at 6 months were lower among those without hypertension (0.43, 0.24–0.78) than among those with hypertension. 

### 3.4. Association between Dietary Habits and Control of Lipid Profiles at 6 Months

Table 4 shows the ORs and 95% CIs for the control of LDL-C, TG, and TC according to each category of the modified MDA items. In the multivariate model, the odds for the control of LDL-C at 6 months was higher among those who consume cholesterol-rich foods <1 time/week (3.27, 1.25–8.57) than among those who consume cholesterol-rich foods ≥4 times/week. In the multivariate model adjusted for age, sex, household income, and marital status, the odds for control of TG at 6 months was higher among those who always drank dairy products (2.96, 1.36–6.44), ate protein-rich foods three times/day (2.94, 1.06–8.10), and followed a regular eating schedule (3.02, 1.30–7.00) than among those who did not drink dairy products, eat protein-rich foods, and follow a regular eating schedule. In the model adjusted for age and sex, the odds for control of TG at 6 months was higher among those who ate fruit everyday (2.15, 1.00–4.67) than among those who did not. The odds for control of TC at 6 months was higher among those who followed a regular eating schedule (3.47, 1.55–7.76) than among those who did not.

## 4. Discussion

Several dietary habits were associated with the control of lipid profiles at 6 months in patients receiving pharmacotherapy for dyslipidemia, whereas other lifestyle factors such as, alcohol consumption, smoking status, and physical activity, were not. In particular, patients who consumed cholesterol-rich foods <1 time/week were more likely to achieve the target LDL-C level at 6 months than those who consumed cholesterol-rich foods ≥4 times/week. Patients who always consume dairy products and protein-rich foods were more likely to have controlled TG levels at 6 months than those who did not consume dairy products and protein-rich foods. Furthermore, patients with a regular eating schedule were more likely to have controlled TG and TC levels at 6 months than those who didn’t have a regular eating schedule.

In this study, patients who consumed cholesterol-rich foods less often were more likely to achieve target LDL-C levels at 6 months. Previous studies have reported that cholesterol intake affects TC and LDL-C levels; however, the effect thereof was less than the impact of saturated fatty acid and trans-fatty acid intake on TC and LDL-C levels [14,15]. A recent meta-regression analysis reported that the LDL-C level increases by 4.58 mg/dL for an increase of 100 mg dietary cholesterol per day [16]. Therefore, several guidelines have recommended limiting daily cholesterol intake to <300 mg for people with high serum cholesterol levels [5,6]. In addition to the daily amount of cholesterol intake, the frequency of eating cholesterol-rich foods influenced LDL-C levels in our analysis. Therefore, dietary counseling based on the frequency of cholesterol-rich food consumption should also be provided to patients with dyslipidemia.

Patients who consumed dairy products daily and protein-rich foods at least three times/day were more likely to have controlled TG levels at 6 months than those who did not consume dairy products and protein-rich foods. In previous epidemiological studies, dairy intake was associated with lower TG levels [17,18,19,20]. The association was significant in healthy individuals as well as in hypertriglyceridemic patients. Potential mechanisms linking dairy product consumption to lower TG levels include bioactive compounds or proteins found in dairy products [21]. Although the exact component responsible for decreasing TG level is difficult to ascertain, dairy proteins are generally insulinotrophic and bind to insulin receptors on vascular and endothelial smooth muscle cells [21,22]. After binding, vasodilation occurs, thereby increasing blood flow and facilitates the clearance and storage of TG and glucose [21,23].

Protein-based diets improve TG levels as well as adiposity and blood pressure [24]. The food items containing protein in the modified MDA include meat, fish, eggs, beans, and tofu. Soy protein, the main component of tofu, reduces serum concentrations of TC, LDL-C, and TG by upregulating hepatic LDL receptors [25]. Furthermore, beans and tofu are sources of plant protein and contain several potential health-related trophic factors, such as isoflavones. It regulates the expression of sterol regulatory element binding protein-2, which participates in cholesterol clearance by detecting intracellular cholesterol concentrations [26]. Mixed findings exist for the association between egg consumption and cholesterol levels [27]. With regard to TG, one randomized intervention study reported that egg consumption reduced plasma TG levels, and another prospective study showed that higher egg consumption in men reduced the risk for high TG [28,29]. The reduction of TG levels could be attributable to decrease in small LDL particles, which are atherogenic due to their long residence time and high susceptibility for subendothelial retention and oxidation [30]. Small LDL particles have been associated with high levels of plasma TG [28,31]. Consumption of fish, which is high in protein and n-3 fatty acids, generally improves levels of TG and HDL-C and platelet aggregation, and red meat intake did not negatively influence TG levels in a recent study [32,33]. Thus, appropriate protein intake could be helpful in the management of dyslipidemia.

In our analysis, having a regular eating schedule was associated with the control of TG and TC levels. Meal regularity influences cardiometabolic health markers, such as lipid profiles, blood pressure, and insulin resistance [34]. Specifically, skipping breakfast resulted in higher TC and LDL-C levels in randomized controlled trials and was associated with elevated TC, TG, LDL-C levels and reduced HDL-C levels in cross-sectional studies [35,36,37,38]. Furthermore, late-night eating was associated with poor lipid profiles [39]. As lipid metabolism is under circadian control in several organs, disruption of molecular clocks may cause lipid dysregulation [40]. Therefore, regular eating should be encouraged in patients with dyslipidemia.

Women, married participants, participants with normal BMI, participants without diabetes, and participants with good compliance with medical treatment were more likely to have controlled lipid profiles at 6 months in this study. One previous study in Korea reported that good adherence to medication was associated with achieving target LDL-C levels, whereas the presence of cardiovascular risk factors, such as diabetes, hypertension, and smoking, were associated with failure to achieve target LDL-C levels [41]. Other studies also emphasized the importance of medical treatment in the control of lipid profiles. Statin treatment before hospitalization was reported as the only independent predictor of achieving target LDL-C levels among patients with acute coronary syndrome, and non-compliance with medical treatment, inadequate monitoring, and incorrect drug regimen were associated with a failure to achieve target LDL-C levels [42,43]. Therefore, compliance with medical treatment should be emphasized for patients with dyslipidemia.

There are some limitations in this study. First, long-term effects of dietary habits on the control of lipid profiles remain uncertain, as our study period was only 6 months. Second, recall bias may have influenced classifications of lifestyle factors, including dietary habits as most classifications in this study were made based on survey responses. Third, our study population was not nationally representative, as participants were enrolled by family physicians of eight hospitals. Geographical bias may exist because these hospitals are located in large metropolitan areas. Despite these limitations, our study has the following strengths. First, our study showed the effect of dietary habits on the control of lipid profiles among patients who are receiving pharmacotherapy for dyslipidemia using real world data. To the best of our knowledge, most previous studies evaluating the association between dietary habits and lipid profiles have been conducted among healthy individuals. In the real world, patients present doubts on dietary modifications once they start pharmacotherapy. Furthermore, physicians do not strongly recommend a diet regimen once patients start medical treatment because lipid profiles will be well controlled under pharmacotherapy. Our results emphasized that patients taking statin can additionally improve lipid profiles depending on their dietary habits. Second, our study showed the effectiveness of the simple questionnaire for evaluating dietary habits. The modified MDA is a simple, 11-item questionnaire, used in the Korean national health screening program. In daily clinical practice, it is difficult for physicians to conduct full nutritional evaluation. However, our study showed that physicians can evaluate nutritional status using a relatively simple tool. As the modified MDA is a validated tool used in the national health screening program, it could be translated to different language and modified considering the food environment of each country. As studies evaluating dietary habits using a validated questionnaire among dyslipidemic patients visiting primary care are scarce, our results emphasize the importance of dietary habits in controlling dyslipidemia and could be applied to primary care fields in the world. In summary, our study clearly demonstrated a significant impact of dietary habits on the control of lipid profiles and provided dietary guides, which can be easily understood by the general public.

## 5. Conclusions

Certain dietary habits were associated with the control of lipid profiles at 6 months in patients receiving pharmacotherapy for dyslipidemia, while other lifestyle factors such as alcohol consumption, smoking status, and physical activity were not associated with the control of lipid profiles in the short term. As healthy dietary habits can be helpful for controlling lipid profiles during pharmacotherapy, these should be emphasized particularly for patients with dyslipidemia. Specifically, patients with dyslipidemia should consume less cholesterol-rich foods, consume more dairy products and protein-rich foods, and follow a regular eating schedule to control lipid profiles.

## Figures and Tables

**Table 1:** Baseline characteristics of the 284 study participants with and without controlled low-density lipoprotein cholesterol (LDL-C) at 6 months.

	Total Participants (*n* = 284)	Participants Who Met the Target LDL-C Goal (*n* = 214)	Participants Who Did Not Meet the Target LDL-C Goal (*n* = 70)	*p*-Value
	*N* (%) or Mean (SD)	*N* (%) or Mean (SD)	*N* (%) or Mean (SD)	
Age (years)				
<60	94 (33.1)	70 (74.5)	24 (25.5)	0.670
60–69	102 (35.9)	81 (79.4)	21 (20.6)	
≥70	88 (31.0)	63 (71.6)	25 (28.4)	
Sex				
Male	123 (43.3)	81 (65.9)	42 (34.1)	0.001
Female	161 (56.7)	133 (82.6)	28 (17.4)	
Household income (KRW/month)				
<200	37 (15.2)	26 (70.3)	11 (29.7)	0.956
200–399	87 (35.7)	66 (75.9)	21 (24.1)	
400–600	66 (27.0)	50 (75.8)	16 (24.2)	
≥600	54 (22.1)	39 (72.2)	15 (27.8)	
Marital status				
Married	227 (82.5)	171 (75.3)	56 (24.7)	0.746
Divorced/Widowed/Separated	38 (13.8)	29 (76.3)	9 (23.7)	
Single	10 (3.6)	8 (80.0)	2 (20.0)	
Alcohol consumption				
No	110 (39.3)	86 (78.2)	24 (21.8)	0.398
Yes	170 (60.7)	125 (73.5)	45 (26.5)	
Smoking status				
No	253 (90.0)	189 (74.7)	64 (25.3)	0.491
Yes	28 (10.0)	23 (82.1)	5 (17.9)	
Physical activity				
Low	109 (39.1)	85 (78.0)	24 (22.0)	0.517
Middle	104 (37.3)	76 (73.1)	28 (26.9)	
High	66 (23.7)	49 (74.2)	17 (25.8)	
BMI (kg/m^2^)				
<23	78 (28.1)	69 (88.5)	9 (11.5)	0.011
23–24.9	84 (30.2)	60 (71.4)	24 (28.6)	
≥25	116 (41.7)	83 (71.6)	33 (28.4)	
Hypertension				
No	153 (53.9)	120 (78.4)	33 (21.6)	0.215
Yes	131 (46.1)	94 (71.8)	37 (28.2)	
Diabetes				
No	227 (80.2)	190 (83.7)	37 (16.3)	< 0.001
Yes	56 (19.8)	23 (41.1)	33 (58.9)	
Compliance in taking the statin at 6 months				
<80%	11 (6.3)	4 (36.4)	7 (63.6)	0.004
≥80%	247 (95.7)	193 (78.1)	54 (21.9)	
SBP (mmHg)	127.7 (12.7)	127.0 (12.9)	130.0 (12.0)	0.084
DBP (mmHg)	76.6 (9.2)	76.4 (9.2)	77.3 (9.0)	0.527
Fasting glucose (mg/dL)	110.4 (23.4)	107.7 (19.7)	118.1 (30.5)	0.012
HbA1c (%)	7.7 (1.0)	7.0 (0.9)	9.1 (2.6)	0.334
TC (mg/dL)	231.7 (32.2)	232.5 (32.1)	229.5 (32.6)	0.503
TG (mg/dL)	146.5 (74.7)	143.6 (77.2)	155.5 (65.8)	0.248
HDL-C (mg/dL)	57.1 (16.9)	58.8 (18.1)	52.0 (11.2)	<0.001
LDL-C (mg/dL)	151.2 (27.7)	150.2 (28.8)	154.4 (23.8)	0.267

LDL-C: low-density lipoprotein cholesterol, SD: standard deviation, KRW: Korean Won, BMI: body mass index, SBP: systolic blood pressure, DBP: diastolic blood pressure, HbA1c: hemoglobin A1c, TC: total cholesterol, TG: triglyceride, HDL-C: high-density lipoprotein cholesterol; P for trend is presented for (2 × n) variables.

**Table 2:** Dietary habits and the control of LDL-C, TG, and TC at 6 months.

		Participants Who Met the Target LDL-C Goal (*n* = 214)	Participants Who Did Not Meet the Target LDL-C Goal (*n* = 70)	P for Trend	Participants with TG < 150 mg/dL (*n* = 194)	Participants with TG ≥ 150 mg/dL (*n* = 90)	P for Trend	Participants with TC < 200 mg/dL (*n* = 186)	Participants with TC ≥ 200 mg/dL (*n* = 98)	P for Trend
		*N* (%)	*N* (%)		*N* (%)	*N* (%)		*N* (%)	*N* (%)	
I drink at least one cup (200 mL) of milk, calcium-fortified soy milk, or other dairy products daily.	No	89 (75.4)	29 (24.6)	0.828	76 (64.4)	42 (35.6)	0.044	80 (67.8)	38 (32.2)	0.454
	Usually	65 (77.4)	19 (22.6)		54 (64.3)	30 (35.7)		56 (66.7)	28 (33.3)	
	Always	59 (73.8)	21 (26.3)		63 (78.8)	17 (21.3)		50 (62.5)	30 (37.5)	
I eat meat, fish, eggs, beans, or tofu at least three times a day.	No	42 (76.4)	13 (23.6)	0.957	34 (61.8)	21 (38.2)	0.090	36 (65.5)	19 (34.5)	0.205
	Usually	128 (74.9)	43 (25.1)		116 (67.8)	55 (32.2)		107 (62.6)	64 (37.4)	
	Always	43 (76.8)	13 (23.2)		43 (76.8)	13 (23.2)		43 (76.8)	13 (23.2)	
I eat vegetables other than Kimchi in every meal.	No	37 (80.4)	9 (19.6)	0.270	34 (73.9)	12 (26.1)	0.763	26 (56.5)	20 (43.5)	0.225
	Usually	103 (76.3)	32 (23.7)		89 (65.9)	46 (34.1)		91 (67.4)	44 (32.6)	
	Always	73 (72.3)	28 (27.7)		70 (69.3)	31 (30.7)		69 (68.3)	32 (31.7)	
I eat a fruit every day (including pulped fruit).	No	37 (80.4)	9 (19.6)	0.483	29 (63.0)	17 (37.0)	0.012	26 (56.5)	20 (43.5)	0.334
	Usually	95 (74.8)	32 (25.2)		78 (61.4)	49 (38.6)		87 (68.5)	40 (31.5)	
	Always	81 (74.3)	28 (25.7)		86 (78.9)	23 (21.1)		73 (67.0)	36 (33.0)	
How often do you eat fried or stir-fried dishes?	≥4/week	8 (61.5)	5 (38.5)	0.541	9 (69.2)	4 (30.8)	0.182	8 (61.5)	5 (38.5)	0.295
	2–3/week	65 (76.5)	20 (23.5)		52 (61.2)	33 (38.8)		52 (61.2)	33 (38.8)	
	<1/week	137 (75.7)	44 (24.3)		130 (71.8)	51 (28.2)		123 (68.0)	58 (32.0)	
How often do you eat foods high in cholesterol (such as pork belly, egg yolk, squid)?	≥4/week	18 (62.1)	11 (37.9)	<0.001	20 (69.0)	9 (31.0)	0.717	19 (65.5)	10 (34.5)	0.134
	2–3/week	63 (64.9)	34 (35.1)		64 (66.0)	33 (34.0)		56 (57.7)	41 (42.3)	
	<1/week	131 (84.5)	24 (15.5)		108 (69.7)	47 (30.3)		110 (71.0)	45 (29.0)	
I eat one of the following items every day: ice cream, cake, sweets, and soft drinks.	Always	50 (76.9)	15 (23.1)	0.637	44 (67.7)	21 (32.3)	0.250	44 (67.7)	21 (32.3)	0.276
	Usually	61 (76.3)	19 (23.8)		48 (60.0)	32 (40.0)		57 (71.3)	23 (28.8)	
	No	100 (74.1)	35 (25.9)		99 (73.3)	36 (26.7)		83 (61.5)	52 (38.5)	
I eat salted seafood, pickled vegetables, or salted fish every day.	Always	14 (73.7)	5 (26.3)	0.507	13 (68.4)	6 (31.6)	0.561	13 (68.4)	6 (31.6)	0.788
	Usually	67 (79.8)	17 (20.2)		55 (65.5)	29 (34.5)		53 (63.1)	31 (36.9)	
	No	131 (73.6)	47 (26.4)		125 (70.2)	53 (29.8)		119 (66.9)	59 (33.1)	
I have a regular eating schedule.	No	36 (78.3)	10 (21.7)	0.960	27 (58.7)	19 (41.3)	0.002	25 (54.3)	21 (45.7)	0.001
	Usually	72 (72.7)	27 (27.3)		59 (59.6)	40 (40.4)		56 (56.6)	43 (43.4)	
	Always	105 (76.6)	32 (23.4)		107 (78.1)	30 (21.9)		105 (76.6)	32 (23.4)	
Among grains, meat/fish/eggs/legumes, vegetables, fruits, and milk products, how many types of foods do you usually eat in a day?	≤3 types	94 (77.0)	28 (23.0)	0.570	85 (69.7)	37 (30.3)	0.594	83 (68.0)	39 (32.0)	0.624
	4 types	87 (73.7)	31 (26.3)		79 (66.9)	39 (33.1)		75 (63.6)	43 (36.4)	
	5 types	28 (73.7)	10 (26.3)		25 (65.8)	13 (34.2)		25 (65.8)	13 (34.2)	
How often do you eat out (excluding meals served at workplace cafeteria)?	≥5/week	14 (70.0)	6 (30.0)	0.107	10 (50.0)	10 (50.0)	0.077	16 (80.0)	4 (20.0)	0.273
	2–4/week	77 (70.6)	32 (29.4)		73 (67.0)	36 (33.0)		61 (56.0)	48 (44.0)	
	<1/week	120 (79.5)	31 (20.5)		108 (71.5)	43 (28.5)		108 (71.5)	43 (28.5)	

LDL-C: low-density lipoprotein cholesterol, TG: triglyceride, TC: total cholesterol.

**Table 3:** Factors associated with the control of lipid profiles at 6 months.

	Control of LDL-C	Control of TG	Control of TC
**OR (95% CI) ^a^**	**OR (95% CI) ^a^**	**OR (95% CI) ^a^**
Age (years)			
<60	1.00	1.00	1.00
60–69	1.20 (0.54–2.66)	0.70 (0.33–1.49)	1.50 (0.70–3.18)
≥70	0.80 (0.35–1.82)	0.80 (0.36–1.80)	0.58 (0.27–1.23)
Sex			
Male	1.00	1.00	1.00
Female	2.46 (1.29–4.68)	3.08 (1.63–5.82)	0.65 (0.36–1.17)
Household income (KRW/month)			
<200	1.00	1.00	1.00
200–399	1.55 (0.59–4.07)	1.30 (0.51–3.33)	0.64 (0.25–1.67)
400–600	1.86 (0.66–5.26)	0.98 (0.37–2.61)	0.55 (0.20–1.50)
≥600	1.44 (0.48–4.36)	1.50 (0.51–4.40)	0.63 (0.22–1.86)
Marital status			
Married	1.00	1.00	1.00
Divorced/Widowed/Separated	0.87 (0.13–5.95)	2.59 (0.39–17.13)	0.49 (0.08–3.20)
Single	0.86 (0.15–4.75)	8.01 (1.40–45.92)	0.47 (0.09–2.56)
Alcohol consumption			
No	1.00	1.00	1.00
Yes	0.83 (0.42–1.65)	0.81 (0.42–1.58)	1.34 (0.71–2.52)
Smoking status			
No	1.00	1.00	1.00
Yes	0.33 (0.10–1.08)	1.28 (0.50–3.24)	1.20 (0.47–3.10)
Physical activity			
Low	1.00	1.00	1.00
Middle	0.69 (0.34–1.40)	0.63 (0.32–1.25)	0.73 (0.38–1.39)
High	0.84 (0.37–1.89)	0.66 (0.31–1.43)	1.04 (0.50–2.17)
BMI (kg/m^2^)			
<23	1.00	1.00	1.00
23–24.9	0.90 (0.45–1.80)	1.32 (0.65–2.70)	0.64 (0.32–1.26)
≥25	2.47 (1.00–6.12)	1.58 (0.72–3.49)	1.09 (0.52–2.29)
Hypertension			
No	1.00	1.00	1.00
Yes	1.18 (0.64–2.16)	1.12 (0.63–2.01)	0.43 (0.24–0.78)
Diabetes			
No	1.00	1.00	1.00
Yes	6.70 (3.24–13.87)	1.01 (0.49–2.06)	0.49 (0.23–1.05)
Compliance in taking the statin at 6 months			
<80%	1.00	1.00	1.00
≥80%	7.27 (1.85–28.58)	1.57 (0.40–6.20)	NA

LDL-C: low-density lipoprotein cholesterol, TG: triglyceride, TC: total cholesterol, OR: odds ratio, CI: confidence interval, KRW: Korean Won, BMI: body mass index, ^a^ Adjusted for age, sex, household income, and marital status.

**Table 4:** Multivariate association between dietary habits and the control of LDL-C, TG, and TC at 6 months.

		Control of LDL-C		Control of TG		Control of TC	
		Model 1	Model 2	Model 1	Model 2	Model 1	Model 2
		OR (95% CI)	OR (95% CI)	OR (95% CI)	OR (95% CI)	OR (95% CI)	OR (95% CI)
I drink at least one cup (200 mL) of milk, calcium-fortified soy milk, or other dairy products daily.	No	1.00	1.00	1.00	1.00	1.00	1.00
	Usually	1.09 (0.55–2.16)	1.03 (0.50–2.14)	1.00 (0.55–1.83)	1.10 (0.56–2.18)	0.86 (0.47–1.59)	0.64 (0.32–1.27)
	Always	0.85 (0.43–1.65)	1.12 (0.54–2.34)	1.99 (1.02–3.90)	2.96 (1.36–6.44)	0.76 (0.41–1.39)	0.58 (0.29–1.16)
I eat meat, fish, eggs, beans, or tofu at least three times a day.	No	1.00	1.00	1.00	1.00	1.00	1.00
	Usually	0.96 (0.46–1.99)	1.04 (0.47–2.30)	1.37 (0.71–2.63)	1.55 (0.74–3.21)	0.84 (0.44–1.62)	0.75 (0.36–1.57)
	Always	1.07 (0.44–2.62)	1.22 (0.44–3.37)	2.24 (0.96–5.24)	2.94 (1.06–8.10)	1.72 (0.74–4.01)	2.11 (0.77–5.75)
I eat vegetables other than Kimchi in every meal.	No	1.00	1.00	1.00	1.00	1.00	1.00
	Usually	0.85 (0.36–1.98)	0.76 (0.29–1.97)	0.74 (0.34–1.59)	0.89 (0.38–2.09)	1.50 (0.75–3.03)	1.71 (0.78–3.75)
	Always	0.65 (0.27–1.55)	0.54 (0.20–1.42)	0.82 (0.37–1.83)	0.87 (0.36–2.09)	1.56 (0.75–3.26)	1.60 (0.71–3.64)
I eat a fruit every day (including pulped fruit).	No	1.00	1.00	1.00	1.00	1.00	1.00
	Usually	0.80 (0.34–1.87)	0.71 (0.28–1.83)	1.01 (0.49–2.07)	0.74 (0.32–1.70)	1.68 (0.83–3.40)	1.66 (0.73–3.77)
	Always	0.64 (0.27–1.52)	0.55 (0.21–1.42)	2.15 (1.00–4.67)	1.61 (0.66–3.93)	1.62 (0.79–3.33)	1.38 (0.61–3.15)
How often do you eat fried or stir-fried dishes?	≥4/week	1.00	1.00	1.00	1.00	1.00	1.00
	2–3/week	2.38 (0.67–8.48)	2.37 (0.58–9.75)	0.78 (0.22–2.85)	0.42 (0.08–2.13)	0.86 (0.25–2.92)	0.81 (0.21–3.17)
	<1/week	1.91 (0.56–6.51)	1.88 (0.47–7.54)	1.19 (0.34–4.26)	0.59 (0.12–2.94)	1.04 (0.32–3.43)	1.02 (0.26–3.93)
How often do you eat foods high in cholesterol (such as pork belly, egg yolk, squid)?	≥4/week	1.00	1.00	1.00	1.00	1.00	1.00
	2–3/week	1.16 (0.48–2.80)	1.12 (0.44–2.81)	0.94 (0.38–2.36)	0.90 (0.33–2.47)	0.64 (0.26–1.57)	0.53 (0.21–1.38)
	<1/week	3.00 (1.24–7.26)	3.27 (1.25–8.57)	0.93 (0.38–2.25)	0.75 (0.28–2.01)	1.25 (0.52–2.97)	1.29 (0.50–3.32)
I eat one of the following items every day: ice cream, cake, sweets, and soft drinks.	Always	1.00	1.00	1.00	1.00	1.00	1.00
	Usually	1.00 (0.45–2.20)	1.00 (0.42–2.38)	0.73 (0.36–1.48)	0.56 (0.25–1.27)	1.11 (0.54–2.30)	0.93 (0.41–2.09)
	No	0.90 (0.44–1.83)	1.03 (0.47–2.25)	1.40 (0.72–2.72)	1.45 (0.67–3.14)	0.72 (0.38–1.36)	0.74 (0.36–1.52)
I eat salted seafood, pickled vegetables, or salted fish every day.	Always	1.00	1.00	1.00	1.00	1.00	1.00
	Usually	1.60 (0.49–5.23)	1.65 (0.36–7.50)	0.99 (0.33–2.96)	1.66 (0.43–6.41)	0.76 (0.26–2.25)	1.28 (0.33–5.01)
	No	1.16 (0.38–3.50)	1.04 (0.25–4.43)	1.26 (0.44–3.60)	2.06 (0.56–7.66)	1.01 (0.36–2.84)	1.47 (0.39–5.58)
I have a regular eating schedule.	No	1.00	1.00	1.00	1.00	1.00	1.00
	Usually	0.82 (0.35–1.92)	0.86 (0.35–2.15)	1.19 (0.57–2.49)	1.32 (0.57–3.07)	0.99 (0.48–2.04)	1.24 (0.55–2.77)
	Always	0.93 (0.41–2.11)	1.07 (0.44–2.60)	2.78 (1.33–5.83)	3.02 (1.30–7.00)	2.62 (1.28–5.37)	3.47 (1.55–7.76)
Among grains, meat/fish/eggs/legumes, vegetables, fruits, and milk products, how many types of foods do you usually eat in a day?	≤3 types	1.00	1.00	1.00	1.00	1.00	1.00
	4 types	0.89 (0.49–1.63)	0.86 (0.44–1.68)	0.94 (0.54–1.64)	0.88 (0.47–1.68)	0.78 (0.45–1.34)	0.74 (0.40–1.38)
	5 types	0.90 (0.38–2.16)	0.85 (0.33–2.18)	1.00 (0.44–2.23)	0.91 (0.36–2.27)	0.68 (0.30–1.51)	0.56 (0.23–1.32)
How often do you eat out (excluding meals served at workplace cafeteria)?	≥5/week	1.00	1.00	1.00	1.00	1.00	1.00
	2–4/week	1.02 (0.35–3.00)	0.93 (0.28–3.08)	1.99 (0.73–5.43)	1.51 (0.49–4.67)	0.32 (0.10–1.03)	0.29 (0.09–1.01)
	<1/week	1.36 (0.45–4.09)	1.22 (0.36–4.22)	2.11 (0.77–5.82)	1.61 (0.51–5.08)	0.55 (0.17–1.83)	0.39 (0.10–1.58)

LDL-C: low-density lipoprotein cholesterol, TG: triglyceride, TC: total cholesterol, OR: odds ratio, CI: confidence interval, Model 1: Adjusted for age and sex, Model 2: Adjusted for age, sex, household income, and marital status.

## Data Availability

All datasets in the current study are not publicly available but are available from the corresponding author on reasonable request.

## Supplementary Materials

The following are available online at https://www.mdpi.com/article/10.3390/nu13113784/s1, Table S1: The modified version of the Mini Dietary Assessment Index for Koreans, Table S2: Baseline characteristics of the 284 study participants with and without controlled TG at 6 months, Table S3: Baseline characteristics of the 284 study participants with and without controlled TC at 6 months.
