# Supplementary material for: Relationship between Dietary Habits and Control of Lipid Profiles in Patients with Dyslipidemia Using Pravastatin
Source: Nutrients. 2021 Oct 25;13(11):3784. doi: 10.3390/nu13113784 (PMC8620026; doi:10.3390/nu13113784)
Supplement: Supplementary file 1 [file nutrients-13-03784-s001.zip › nutrients-1429849-supplementary.pdf]

Supplementary Materials

Table S1. The modified version of the Mini Dietary Assessment Index for Koreans.

Questions	Response options
1. I drink at least one cup (200 mL) of milk, calcium-fortified soy milk, or other daily products daily.	· Always<br>· Usually<br>· No
2. I eat meat, fish, eggs, beans, or tofu at least three times a day.	· Always<br>· Usually<br>· No
3. I eat vegetables other than Kimchi in every meal.	· Always<br>· Usually<br>· No
4. I eat a fruit everyday (including pulped fruit).	· Always<br>· Usually<br>· No
5. How often do you eat fried or stir-fried dishes?	· ≥ 4/week<br>· 2–3/week<br>· < 1/week
6. How often do you eat foods high in cholesterol (pork belly, egg yolk, squid)?	· ≥ 4/week<br>· 2–3/week<br>· < 1/week
7. I eat one of the following items every day: ice cream, cake, sweets, and soft drinks.	· Always<br>· Usually<br>· No
8. I eat salted seafood, pickled vegetables, or salted fish every day.	· Always<br>· Usually<br>· No
9. I have a regular eating schedule.	· Always<br>· Usually<br>· No
10. Among grains, meat/fish/eggs/legumes, vegetables, fruits, and milk products, how many types of foods do you usually eat in a day?	· 5 types<br>· 4 types<br>· ≤ 3 types
11. How often do you eat out (excluding meals served at workplace cafeteria)?	· ≥ 5/week<br>· 2–4/week<br>· < 1/week

Table S2. Baseline characteristics of the 284 study participants with and without controlled TG at 6 months.

	**Participants with TG < 150 mg/dL at 6 months (n = 194)**	**Participants with TG ≥ 150 mg/dL at 6 months (n = 90)**	**p-value**
	N (%) or Mean (SD)	N (%) or Mean (SD)	
Age (years)			
< 60	66 (70.2)	28 (29.8)	0.644
60–69	69 (67.6)	33 (32.4)	
≥ 70	59 (67.0)	29 (33.0)	
Sex			
Male	70 (56.9)	53 (43.1)	< 0.001
Female	124 (77.0)	37 (23.0)	
Household income (KRW/month)			
< 200	22 (59.5)	15 (40.5)	0.377
200–399	61 (70.1)	26 (29.9)	
400–600	40 (60.6)	26 (39.4)	

≥ 600	40 (74.1)	14 (25.9)	
Marital status			
Married	165 (72.7)	62 (27.3)	0.001
Divorced/Widowed/Separated	21 (55.3)	17 (44.7)	
Single	3 (30.0)	7 (70.0)	
Alcohol consumption			
No	78 (70.9)	32 (29.1)	0.599
Yes	115 (67.6)	55 (32.4)	
Smoking status			
No	177 (70.0)	76 (30.0)	0.088
Yes	15 (53.6)	13 (46.4)	
Physical activity			
Low	80 (73.4)	29 (26.6)	0.357
Middle	66 (63.5)	38 (36.5)	
High	45 (68.2)	21 (31.8)	
BMI (kg/m^2)			
< 23	60 (76.9)	18 (23.1)	0.025
23–24.9	60 (71.4)	24 (28.6)	
≥ 25	72 (62.1)	44 (37.9)	
Hypertension			
No	108 (70.6)	45 (29.4)	0.443
Yes	86 (65.6)	45 (34.4)	
Diabetes			
No	159 (70.0)	68 (30.0)	0.201
Yes	34 (60.7)	22 (39.3)	
Compliance in taking the statin at 6 months			
< 80%	7 (63.6)	4 (36.4)	0.745
≥ 80%	170 (68.8)	77 (31.2)	
SBP (mmHg)	126.8 (12.6)	129.6 (12.8)	0.087
DBP (mmHg)	76.5 (9.2)	76.9 (9.2)	0.769
Fasting glucose (mg/dL)	109.5 (22.2)	112.2 (25.7)	0.390
HbA1c (%)	7.2 (1.0)	8.5 (2.3)	0.554
TC (mg/dL)	233.5 (31.6)	227.8 (33.2)	0.165
TG (mg/dL)	123.0 (57.6)	198.4 (81.8)	< 0.001
HDL-C (mg/dL)	60.4 (17.4)	50.0 (13.4)	< 0.001
LDL-C (mg/dL)	152.6 (27.0)	148.2 (29.2)	0.217

TG: triglyceride, SD: standard deviation, KRW: Korean Won, BMI: body mass index, SBP: systolic blood pressure, DBP: diastolic blood pressure, HbA1c: hemoglobin A1c, TC: total cholesterol, HDL-C: high-density lipoprotein cholesterol, LDL-C: low-density lipoprotein cholesterol,.P for trend is presented for (2 x n) variables.

Table S3. Baseline characteristics of the 284 study participants with and without controlled TC at 6 months.

	Participants with TC < 200 mg/dL at 6 months (n = 186)	Participants with TC ≥ 200 mg/dL at 6 months (n = 98)	p-value
	N (%) or Mean (SD)	N (%) or Mean (SD)	
Age (years)			
< 60	51 (54.3)	43 (45.7)	0.030
60–69	74 (72.5)	28 (27.5)	
≥ 70	61 (69.3)	27 (30.7)	
Sex			
Male	86 (69.9)	37 (30.1)	0.208
Female	100 (62.1)	61 (37.9)	
Household income (KRW/month)			
< 200	28 (75.7)	9 (24.3)	0.233
200–399	57 (65.5)	30 (34.5)	
400–600	41 (62.1)	25 (37.9)	
≥ 600	34 (63.0)	20 (37.0)	
Marital status			

Married	149 (65.6)	78 (34.4)	0.830
Divorced/Widowed/Separated	25 (65.8)	13 (34.2)	
Single	7 (70.0)	3 (30.0)	
Alcohol consumption			
No	74 (67.3)	36 (32.7)	0.796
Yes	111 (65.3)	59 (34.7)	
Smoking status			
No	168 (66.4)	85 (33.6)	0.835
Yes	18 (64.3)	10 (35.7)	
Physical activity			
Low	72 (66.1)	37 (33.9)	0.974
Middle	67 (64.4)	37 (35.6)	
High	44 (66.7)	22 (33.3)	
BMI (kg/m^2)			
< 23	54 (69.2)	24 (30.8)	0.860
23–24.9	52 (61.9)	32 (38.1)	
≥ 25	78 (67.2)	38 (32.8)	
Hypertension			
No	91 (59.5)	62 (40.5)	0.024
Yes	95 (72.5)	36 (27.5)	
Diabetes			
No	142 (62.6)	85 (37.4)	0.059
Yes	43 (76.8)	13 (23.2)	
Compliance in taking the statin at 6 months			
< 80%	0 (0.0)	11 (100.0)	< 0.001
≥ 80%	171 (69.2)	76 (30.8)	
SBP (mmHg)	127.4 (13.3)	128.2 (11.6)	0.627
DBP (mmHg)	76.3 (9.6)	77.3 (8.3)	0.354
Fasting glucose (mg/dL)	111.9 (21.1)	107.2 (27.3)	0.137
HbA1c (%)	6.3 (1.0)	10.8 (3.3)	0.173
TC (mg/dL)	224.3 (30.1)	245.8 (31.3)	< 0.001
TG (mg/dL)	146.5 (79.9)	146.6 (63.5)	0.992
HDL-C (mg/dL)	55.2 (16.4)	60.8 (17.5)	0.008
LDL-C (mg/dL)	146.3 (28.1)	160.7 (24.3)	< 0.001

TG: triglyceride, SD: standard deviation, KRW: Korean Won, BMI: body mass index, SBP: systolic blood pressure, DBP: diastolic blood pressure, HbA1c: hemoglobin A1c, TC: total cholesterol, HDL-C: high-density lipoprotein cholesterol, LDL-C: low-density lipoprotein cholesterol, P for trend is presented for (2 x n) variables.
